# Protein charge distribution in proteomes and its impact on translation

**DOI:** 10.1371/journal.pcbi.1005549

**Published:** 2017-05-22

**Authors:** Rodrigo D. Requião, Luiza Fernandes, Henrique José Araujo de Souza, Silvana Rossetto, Tatiana Domitrovic, Fernando L. Palhano

**Affiliations:** 1 Programa de Biologia Estrutural, Instituto de Bioquímica Médica Leopoldo de Meis, Universidade Federal do Rio de Janeiro, Rio de Janeiro, Rio de Janeiro, Brazil; 2 Programa de Pós-Graduação em Informática, Universidade Federal do Rio de Janeiro, Rio de Janeiro, Rio de Janeiro, Brazil; 3 Departamento de Virologia, Instituto de Microbiologia Paulo de Góes, Universidade Federal do Rio de Janeiro, Rio de Janeiro, Brazil; University of Missouri, UNITED STATES

## Abstract

As proteins are synthesized, the nascent polypeptide must pass through a negatively charged exit tunnel. During this stage, positively charged stretches can interact with the ribosome walls and slow the translation. Therefore, charged polypeptides may be important factors that affect protein expression. To determine the frequency and distribution of positively and negatively charged stretches in different proteomes, the net charge was calculated for every 30 consecutive amino acid residues, which corresponds to the length of the ribosome exit tunnel. The following annotated and reviewed proteins in the UniProt database (Swiss-Prot) were analyzed: 551,705 proteins from different organisms and a total of 180 million protein segments. We observed that there were more negative than positive stretches and that super-charged positive sequences (i.e., net charges ≥ 14) were underrepresented in the proteomes. Overall, the proteins were more positively charged at their N-termini and C-termini, and this feature was present in most organisms and subcellular localizations. To investigate whether the N-terminal charges affect the elongation rates, previously published ribosomal profiling data obtained from *S*. *cerevisiae*, without translation-interfering drugs, were analyzed. We observed a nonlinear effect of the charge on the ribosome occupancy in which values ≥ +5 and ≤ -6 showed increased and reduced ribosome densities, respectively. These groups also showed different distributions across 80S monosomes and polysomes. Basic polypeptides are more common within short proteins that are translated by monosomes, whereas negative stretches are more abundant in polysome-translated proteins. These findings suggest that the nascent peptide charge impacts translation and can be one of the factors that regulate translation efficiency and protein expression.

## Author summary

Which factors shape the sequence of amino acids that will form a protein? The biochemical features of amino acids, such as their charge and hydrophobicity, are important drivers of protein tridimensional folding, which creates interaction sites for binding other molecules and directs proteins to specific cellular compartments. These features all impact the activity of the proteins after they are produced. Another less obvious factor that influences the protein’s primary structure may be how efficiently a given amino acid sequence is produced by the ribosome. It is known that a repetitive stretch of positively charged amino acids may interact with the negative charges in the ribosome exit tunnel, slowing, or even halting, translation. By analyzing the charge of protein stretches in different organisms, we observed that proteins tend to present positively charged stretches at their extremities, and high charge values can slow (for positive charges) or speed (for negative charges) translation. An interesting consequence of this trend is that proteins that are translated in high quantities by several ribosomes at the same RNA (polysomes) tend to have more negatively charged stretches than proteins that are translated by a single ribosome per RNA (monosomes).

## Introduction

The translation of messenger RNAs is the final step in translating the genetic code from DNA to proteins and is an essential, energetic expensive reaction[1]. The translation process is regulated by different mechanisms, primarily at the initiation phase. These mechanisms include initiation factor levels and posttranslational modifications, mRNA accessibility, microRNA activity and trans-acting protein activity[1]. The termination phase may also be subject to regulation by permitting frame-shifting or the insertion of amino acids where the translation should end[1]. The elongation rate can be influenced by the messenger RNA secondary structure[1], rare codon usage[2,3] or nascent polypeptide charge[4] and appears to be tightly coupled to co-translational folding[5]. The translation efficiency is directly affected by the elongation rate as slow rates can, in certain cases, lead to ribosome stalling and interruption of translation[6]. The elongation and initiation rates can also determine whether the transcripts are translated in the monosomes or polysomes[7].

Most nascent polypeptides are synthesized in the ribosomal exit tunnel. This tunnel provides a passage that is approximately 100 Å long through the large subunit from the peptidyl transferase center to the cytosol[8] and houses approximately 30 amino acids residues of the nascent polypeptide[9]. The tunnel is composed primarily of nucleic acids and contains two proteins (L4 and L17) that line the tunnel’s interior[9]. Because nucleic acids are the major constituents, the electrostatic potential is negative, ranging from -8 mV to -22 mV[10]. Experiments using recombinant proteins designed to function as molecular tape measures have shown that positively charged sequences (rich in arginine/lysine residues) are able to arrest translation, while neutral or negatively charged sequences promote faster elongation rates[4]. This finding indicates that positively charged residues are able to interact with the ribosome exit tunnel and possibly promote ribosome stalling.

The effect of the nascent polypeptide composition on the translation rates has also been investigated using the ribosome profile technique, which is based on the deep sequencing of ribosome-protected mRNA fragments during translation[11]. Using this technique, there are certain contradictory conclusions that appear to originate from the use of translational inhibitors, such as cycloheximide, which are usually added to stabilize the ribosomes prior to the cell lysis[11–14]. When performed in the absence of such drugs, experiments tend to consistently show that positively charged sequences are able to promote slower elongation rates[14].

If the nascent polypeptide charge is an important factor affecting ribosomal translation efficiency, one might expect that the distribution of charges across a protein sequence should be shaped not only by structural and functional pressures but also by their effect on protein synthesis. It has already been demonstrated that there is a higher concentration of positively charged residues at the N- and C-termini of proteins[15–17]. Tentative explanations for this observation involve either the charge effect on translation or the importance of the positive stretches to the protein structure. An early work using the technique of ribosome profiling to assess the speed of translation showed that the translation rates were slower at the N-termini of proteins. The authors suggested that the so-called ribosomal ramp is an inherent component of the translation initiation and is important for organizing polysomal translation and avoiding jams[18]. This model could explain the enrichment of positive charges at the N-terminus because this could be one of the factors that causes the increase in the ribosomal density in this area. However, many aspects of this hypothesis have been debated in the literature[19,20]. Even the functional meaning of a ribosomal ramp has been questioned since the observed increase in the ribosome density at the beginning of the transcripts appears to be induced by the addition of cycloheximide during the sample preparation and, therefore, cannot be considered a natural characteristic of ribosomal translation[16,17].

An alternative hypothesis was offered by Charneski and Hurst[17], who suggested that the enrichment of positively charged residues at the protein extremities could be explained by the presence of positive patches adjacent to the transmembrane protein segments. These basic domains lie on the cytoplasmic side and control the protein orientation by electrostatic interactions with the inner layer of phospholipids[17]. By analyzing the first 30 amino acids of *E*. *coli* and *S*. *cerevisiae* proteins, the authors found that basic N-termini are present in membrane proteins but not in cytoplasmic proteins. The authors also demonstrated that the specific distribution of Arg or Lys in the N- and C- termini could be caused by the natural tendency for positively charged patches to occur close to the protein extremities. Taken together, these data indicate that there is no consensus regarding whether the translation efficiency is a factor that influences the distribution of charges in the primary structure of proteins.

In this study, 551,705 proteins from different organisms from *E*. *coli* to *H*. *sapiens* were analyzed to search for charged sequences and their biological relevance. We found that there are more negatively charged sequences than positively charged sequences, and extremely positively charged sequences are underrepresented in most proteomes. Proteins tend to concentrate their positively charged sequences at their N- and C-termini, and this feature appears to be strongly conserved across different organisms and subcellular locations, including cytoplasmic proteins. By analyzing previously published ribosome-profiling analyses of *S*. *cerevisiae* that were performed without cycloheximide, we concluded that the nascent polypeptide charge can modulate the ribosome occupancy, but this effect is only apparent in peptides with a net positive N-terminal charge ≥ +5 and ≤ -6. Finally, using ribosome-profiling data from different monosomal/polysomal population, it is possible to observe that the N-terminal net charges are associated with monosomal translation, whereas neutral and negative stretches are more common in the N-termini in proteins that are translated by polysomes. Moreover, because most proteins contain basic stretches within the net-charge range that have little effect on the translation rate, our analysis suggests that a widespread ribosomal ramp is not important for organizing the polysomal translation. These data corroborate the notion that the translation rate can act as a selection force to shape the charge distribution along the primary structures of proteins.

## Results

### Extremely positively charged protein segments are underrepresented in most proteomes

To determine the charge frequency of protein segments in various proteomes, we developed a program that is able to screen the sequence of a given protein and calculate the net charge of every 30-amino acid segment, which is the estimated polypeptide length that occupies the ribosome exit tunnel[9].

The net charge was calculated by considering the protonation state of the amino acids according to their pKa values at a physiological pH of 7.4. Positively charged residues (lysine and arginine) were considered +1; negatively charged residues (glutamic and aspartic acid) were considered -1; and all other residues were considered 0. For the sake of simplicity, we eliminated the C- and N-termini charges and disregarded the cysteine contribution (charge at 7.4 = − 0.085). Histidine deserved special attention since its pKa value can vary from 2.4 to 9.2 depending on the chemical environment within the protein[21,22]. In an unfolded peptide of five amino acids (Ala-Ala-His-Ala-Ala), the pKa of histidine was determined to be 6.5[21,22]. This finding means that at physiological pH values of 7.0–7.4, histidines are more likely to be uncharged. However, because previous analyses considered histidine a positively charged amino acid[15,17], calculations were performed by considering both His +1 and His 0.

**Fig 1:**
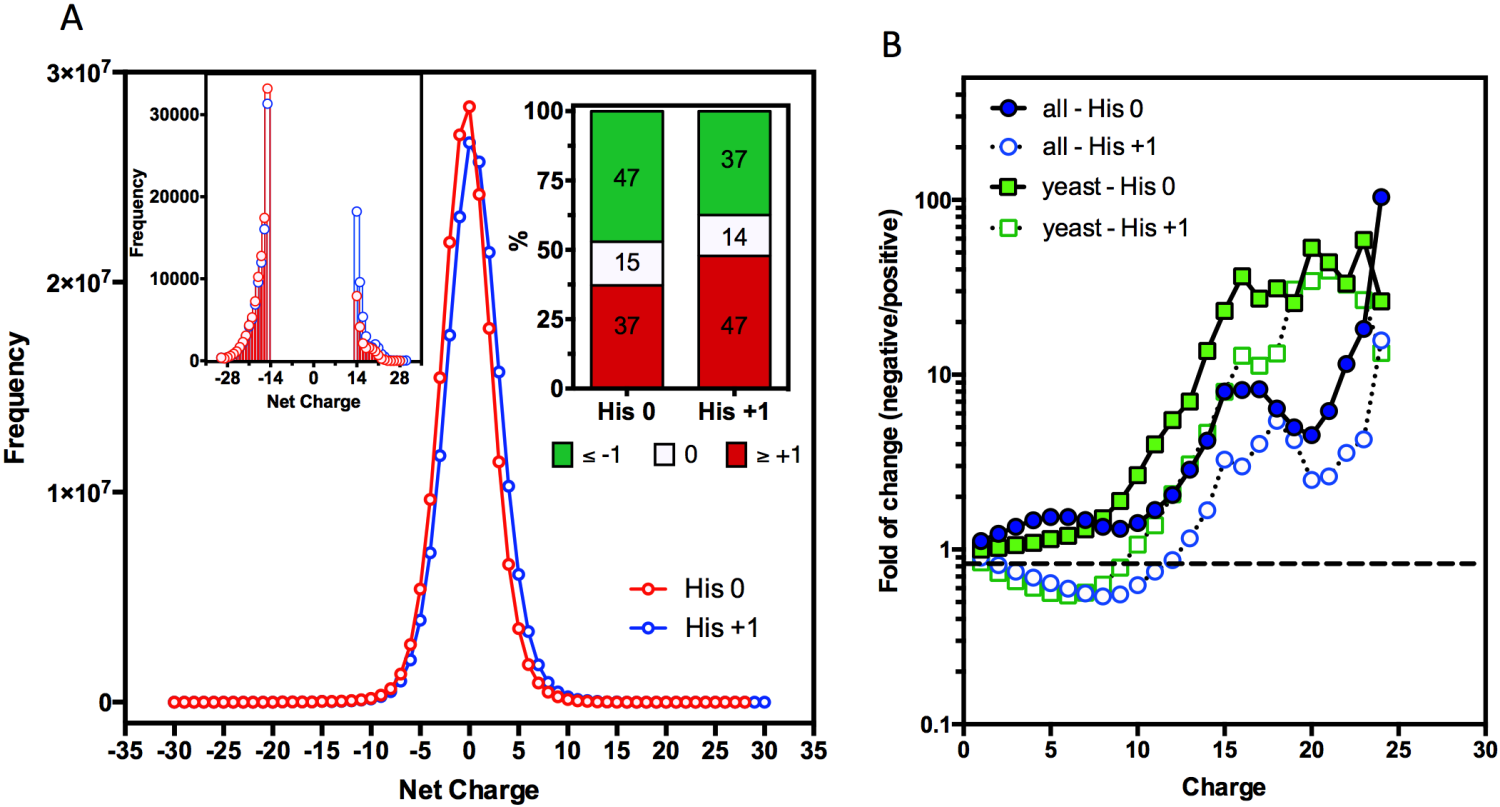
Supercharged positive sequences are underrepresented in most proteomes. **A.** Net-charge frequency histogram of the amino-acid segments in all 551,705 proteins from the SwissProt database with the histidine charge considered 0 (red line) and +1 (blue line). When histidine was considered 0, it is possible to observe that 47% of the sequences were negatively charged, 15% of the sequences were neutral and 37% of the sequences were positively charged. When histidine was considered +1, 37% of the sequences were negatively charged, 14% of the sequences were neutral and 47% of the sequences were positively charged (upper-right inset). Super charged positive sequences (+14 or more) were less frequent than super charged negative sequences, even when histidine was considered +1 (upper-left inset). **B.** Net charge ratio between the negative and positive sequences shows a steep increase around charge 14. Analysis of the *S*. *cerevisiae* proteome (6,721 proteins, green squares) showed a pattern that was similar to that in all 551,705 proteins (blue circles). Analyses performed with histidine considered +1 showed similar results (empty symbols).

The annotated and reviewed proteins in the UniProt database (Swiss-Prot) were analyzed, including 551,705 proteins from different organisms and 180 million 30-amino acid segments. The histidine charge affected the distribution of the net charge in the protein segments. As shown in Fig 1A, the presence of charged histidines caused a shift in the frequency distribution curve toward positive net-charge values, and as shown in Fig 1A (right inset), the charged histidines increased the proportion of positively charged sequences relative to the negatively charged segments.

Another observation was that super-charged segments with positive net-charges > +14 appeared less frequently than segments with negative net-charges < -14 (Fig 1A, left inset). This feature can be better observed in Fig 1B, in which the ratio of the number of 30-amino acid sequences that possess equal but opposite net-charge values (negative/positive) was determined. If the number of negative and positive stretches of a given charge value is approximately the same, the result is one. Values greater than one reflect a ratio of negative over positive stretches, and values smaller than one reflect the opposite (Fig 1B). The plot shows that the negatively charged sequences become more abundant than the positively charged stretches at net-charge values greater than 10. Furthermore, the histidine charge primarily affects the sequences with moderate net-charge values (i.e., 5–10). When His is considered +1, the average ratio of these charge values shifts toward positively charged sequences; however, at net-charge values greater than 10, the negatively charged segments become more frequent regardless of the histidine charge. In most organisms, the intracellular pH, except in certain specific organelles, varies between 7.0 and 7.5[23]. Therefore, a net charge of 0 was adopted to more closely resemble the physiological scenario in all subsequent analyses.

### Proteins from different organelles concentrate positive charges at their N- and C- termini

**Fig 2:**
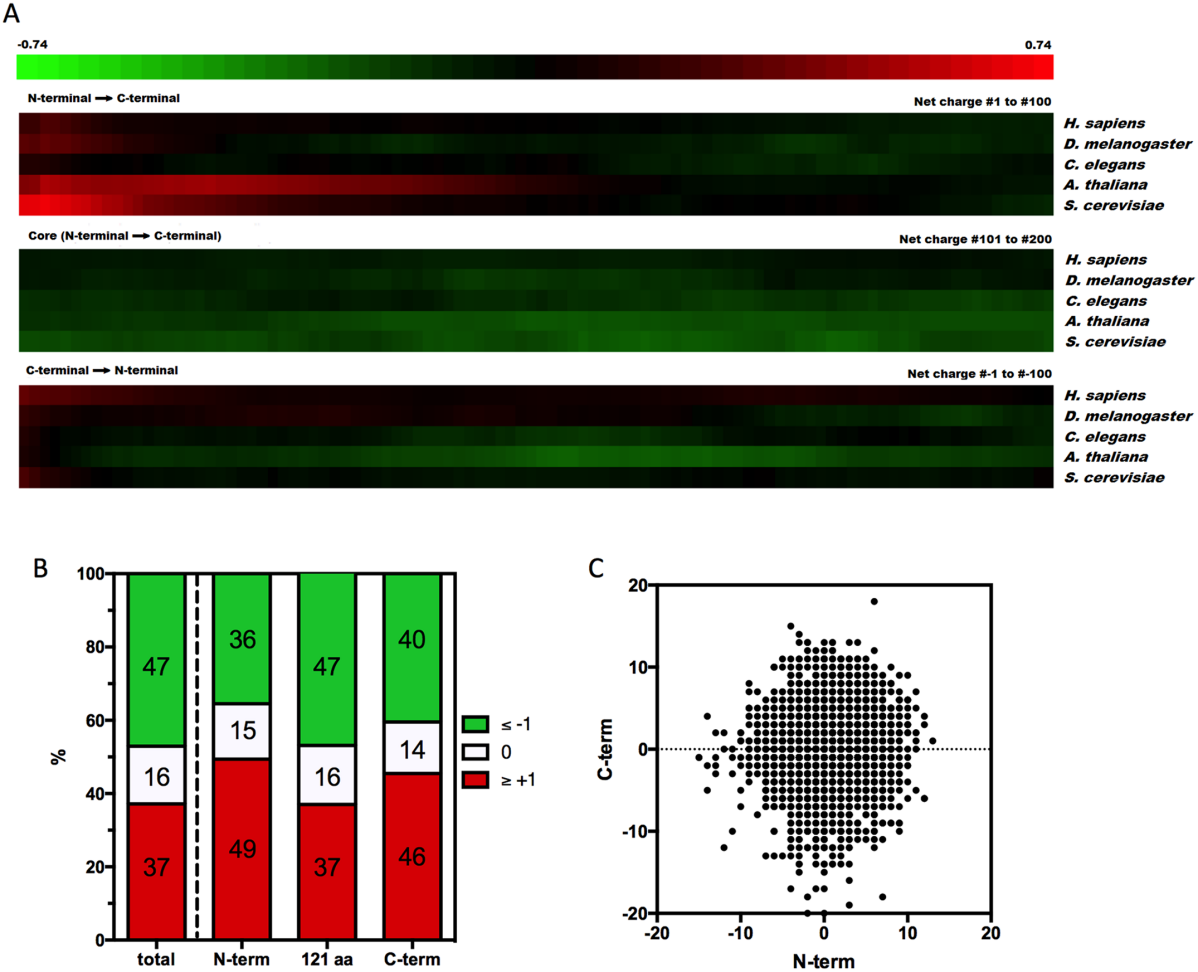
Positive charged sequence concentrations at the N- and C-termini. **A.** Heat map of the *H*. *sapiens* (20,177 proteins), *D*. *melanogaster* (3,341 proteins), *C*. *elegans* (3,743 proteins), *A*. *thaliana* (14,791 proteins) and *S*. *cerevisiae* (6,721 proteins) proteome net charge distribution. Red tiles represent the positively charged sequences, black tiles represent the neutral sequences and green tiles represent the negatively charged sequences. The number +/- 0.74 is the mean net charge of the organisms. Our analyses reveal a strong concentration of net positive charges at the N-termini (upper panel, net charges from amino acids 1 to 30 until 100 to 129), a concentration of net negative charges at their core (middle panel, net charges from amino acids 130 to 159 until 230 to 259) and a smaller, but still present, concentration of positive net charges at the C-termini (bottom panel, net charges from amino acids -129 to -100 until -30 to -1). **B.** Frequency of the net charges in all 551,705 proteins separated by 30 amino acid segments of the primary sequence. It is possible to observe that the N-terminal regions show 49% net positively charged segments, 15% neutral segments and 36% net negatively charged segments. The core regions display 37% net positively charged segments, 16% neutral segments and 47% net negatively charged segments. The c-terminal regions display 46% net positively charged segments, 14% neutral segments and 40% net negatively charged segments. **C**. The net charge of the first 30 residues (abscissa) of each of the 6,721 proteins from *S*. *cerevisiae* is plotted against the net charge of the last 30 residues of the same protein (ordinate). There is a small but positive correlation between the N-terminal and C-terminal charges (P < 0.0001, R^2^ = 0.006479) in *S*. *cerevisiae*.

Previous analyses by other groups indicated that the N- and C- termini of proteins tend to be enriched with positively charged residues. To determine whether these regions contain more positively charged stretches, the net charges of 30-residue segments along the primary structures of proteins were investigated. As shown in Fig 2A, a heat map was used to depict the charge distribution at the N-termini (i.e., first 100 net charges); core (i.e., net charges 101–200) and C-termini (i.e., last 100 net charges) of proteins. The color of each tile corresponds to the net charge of 30 consecutive amino acids (i.e., first tile 1–30, second tile 2–31, etc.). Consistently with other analyses [15–17], it is possible to observe that the N- and C-terminal regions tend to be enriched with positive charges, while the core of most sequences contains more negative and neutral segments (Fig 2A). Fig 2B shows that 49 and 46% of the sequences are positively charged at their first and last 30 amino acid residues, respectively, whereas 63% of the segments from the middle of the primary sequence are neutral or negatively charged. Fig 2C shows the correlation between the net-charge of the first and last 30 residues in each of the 6,663 proteins of the *S*. *cerevisiae* proteome. There was a positive correlation (P < 0.0001), but this was observed for only a small number of proteins (R^2^ = 0.006479) (Fig 2C).

**Fig 3:**
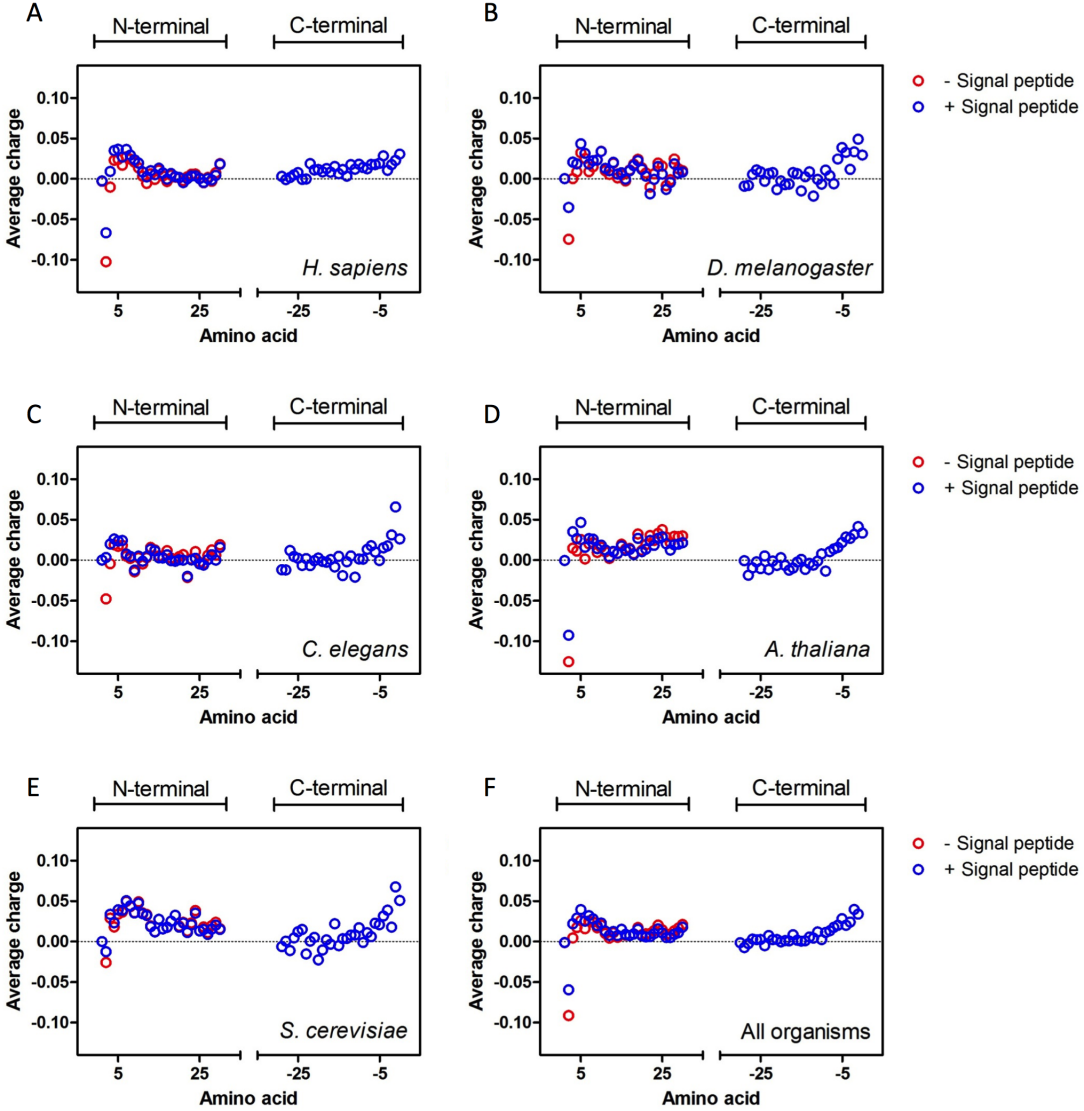
Average charge of each residue in the first and last 30 amino acids of all recorded proteins from each species. **A.** *H*. *sapiens*, **B.** *D*. *melanogaster*, **C.** *C*. *elegans*, **D.** *A*. *thaliana*, **E.** *S*. *cerevisiae* proteomes and **F.** these five proteomes combined. Blue circles represent whole proteomes (*H*. *sapiens* 20,177, *D*. *melagnogaster* 3,341, *C*. *elegans* 3,743, *A*. *thaliana* 14,791 and *S*. *cerevisiae* 6,721 proteins), whereas red circles represent proteomes minus proteins that possess signal peptides (*H*. *sapiens* 16,717 proteins, *D*. *melanogaster* 2,883 proteins, *C*. *elegans* 3,232 proteins, *A*. *thaliana* 12,599 proteins, and *S*. *cerevisiae* 6,382 proteins). Our N-terminal analyses show conservation of a neutral residue at position number 1, a negatively charged residue at position number 2 and a concentration of positively charged residues from approximately position number 3 to 10. Our C-terminal analyses show conservation of positively charged residues from approximately amino acids -15 to -10 onwards.

The subsequent analysis provided information regarding the charge at each position in the first and last 30 amino acid residues of proteins. (Fig 3A–3F). In this analysis, each data point represents the average charge of the residues that occupy a specific position in the protein sequence. For example, the first point is always zero since it represents the starting methionine. A similar pattern was observed in all organisms analyzed. At the N-terminus, the second amino acid residue tends to be negatively charged, followed by positive charges up until amino acid number ten; from amino acid 11 to 30, the average charge tends to be only slightly positive (Fig 3). The C-terminal analysis shows a different distribution in which there is a steep increase in positive charges around the last ten to fifteen amino acids (Fig 3). Since signal peptides, which are responsible for targeting proteins to the endoplasmic reticulum and, consequently, to the secretory pathway, are known to contribute positive charges to the N-terminus[24], the analysis was repeated after excluding proteins that were identified as having signal peptides in the Uniprot database (Fig 3, red symbols). The same charge distribution profile was observed with both datasets (Fig 3).

Certain aspects of the specific distribution of the amino acid charges can be readily explained. The first amino acid tends to be neutral, which is logical because the first amino acid is usually a methionine residue (Fig 3). The second amino acid tends to be negative (Fig 3). This observation can be explained by the Kozak consensus sequence, which predicts a high prevalence of G after the initiation codon AUG[25]. Every codon that starts with G codes for either a neutral or a negatively charged amino acid, which explains this observed phenomenon. When proteins with secretion signal peptides are removed from the analysis, the average charge of the second amino acid is even lower. This observation can be explained by the common presence of lysine residues at amino acid position two in the signal peptides, which tend to be rich in positively charged residues[24]. An increased occurrence of basic peptides in the C-termini of proteins was also observed. This result is consistent with previous analyses that show that the basic amino acids in the last two positions before the stop codon can greatly enhance the translation termination efficiency[26,27]. However, these observations alone cannot account for the general and conserved tendency of the positively charged protein segments to encompass dozens of residues at the N- or C-termini of proteins (Fig 2A).

**Fig 4:**
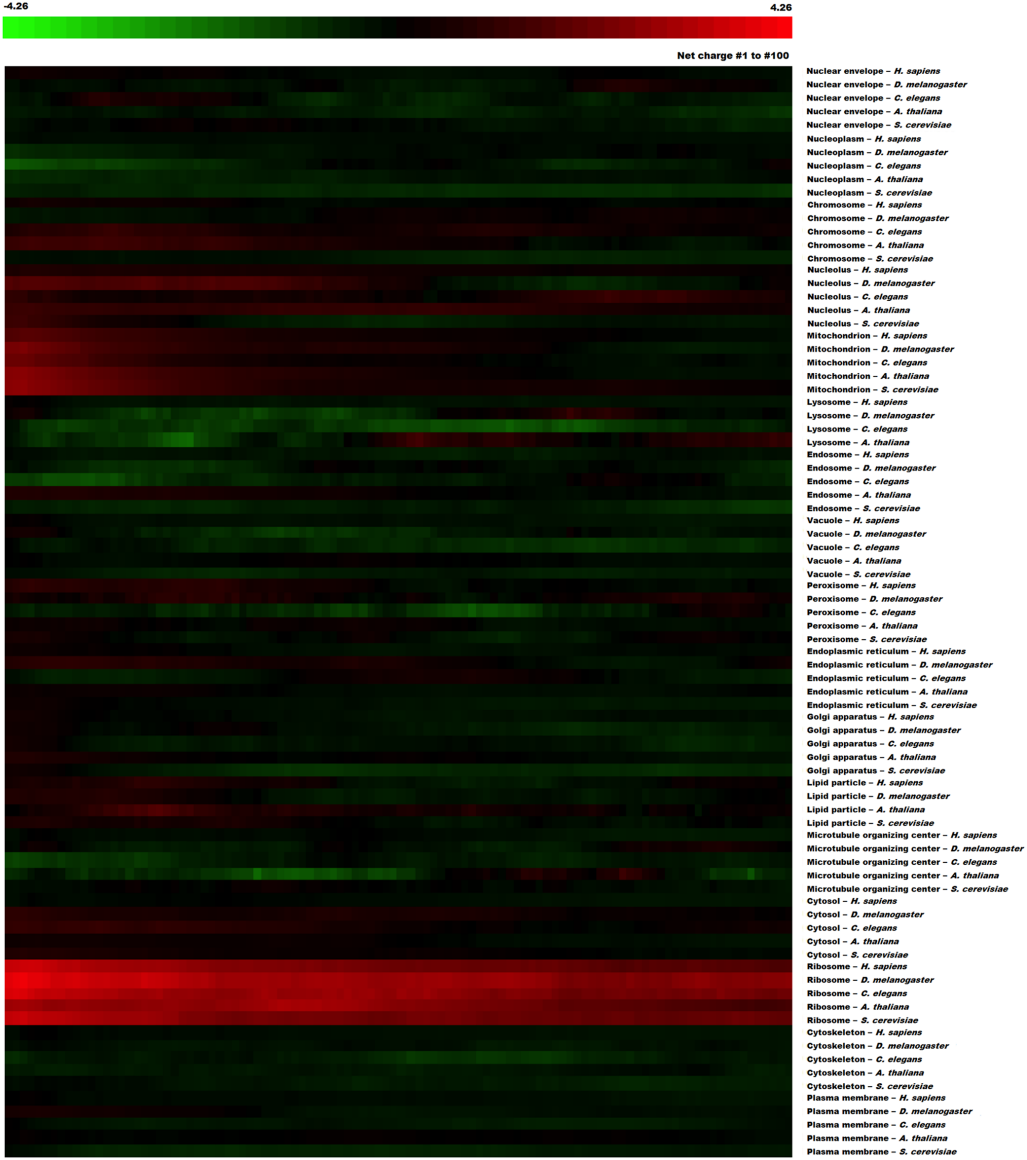
N-terminal positive charge concentration of proteins from different subcellular locations. Heat map for *H*. *sapiens*, *D*. *melanogaster*, *C*. *elegans*, *A*. *thaliana* and *S*. *cerevisiae* proteomes showing the average net charge distribution (net charges from amino acids 1 to 30 until 100 to 129) divided into 17 different subcellular locations. Red tiles represent segments with a net positive charge, black tiles represent neutral segments and green tiles represent segments with a net negative charge. N-terminal positive charge concentration is conserved among most subcellular locations, except for endosomes, lysosomes, vacuoles and nucleoplasm proteins.

To assess whether the positively charged segments occur more frequently in membrane proteins, we sorted the *H*. *sapiens*, *D*. *melanogaster*, *C*. *elegans*, *A*. *thaliana* and *S*. *cerevisiae* protein databases according to their subcellular localization based on Gene Ontology (GO)[28] and calculated the average charge distribution of the first (Fig 4) and last (S1 Fig) 100 residues from proteins from each organelle. The positively charged N-termini could be observed in different organelles and cytoplasmic proteins from different species. Even when the average net charge of a given type of organelle was highly positive (i.e., nucleolus, mitochondria and ribosomes), the N-terminus showed an even higher concentration of positively charged residues (Fig 4, S2 Fig).

**Fig 5:**
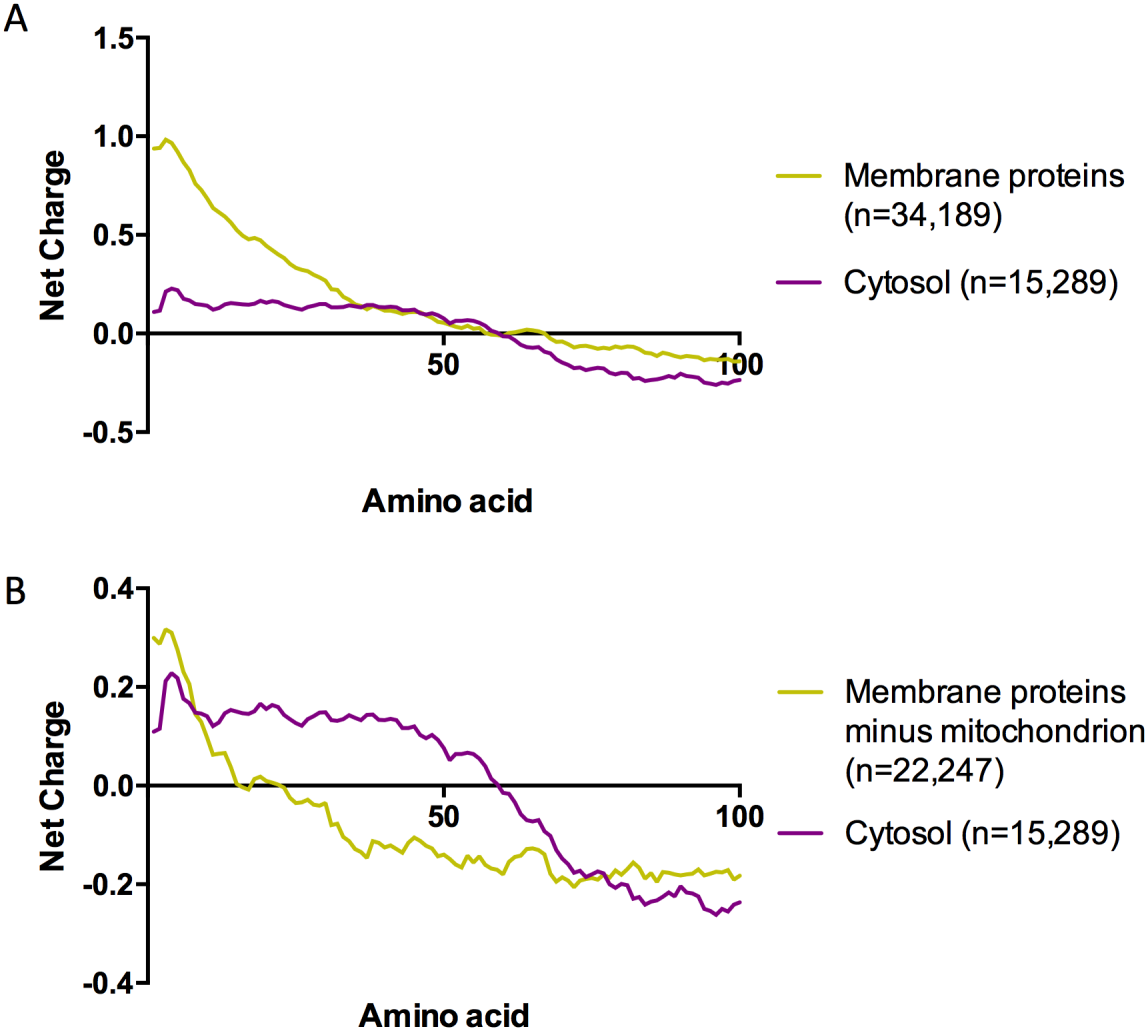
N terminal net charge distribution in cytosolic and membrane proteins from different organelles. **A.** Proteins from *H*. *sapiens*, *D*. *melanogaster*, *C*. *elegans*, *A*. *thaliana* and *S*. *cerevisiae* localized in different organelles (mitochondria, endoplasmic reticulum, Golgi apparatus and vacuoles) were compared with proteins from the cytoplasm. The net charge of consecutive windows of 30 amino acids were calculated up to amino acid number 100. **B.** Same as that shown in A, but the mitochondrial proteins were removed from the “membrane” dataset.

Fig 5 shows the net charge of consecutive stretches of 30 amino acids (i.e., 1–30; 2–31) from proteins in *H*. *sapiens*, *D*. *melanogaster*, *C*. *elegans*, *A*. *thaliana* and *S*. *cerevisiae* localized in the cytoplasm and organelles that are enriched with membrane proteins (mitochondria, endoplasmic reticulum, Golgi apparatus and vacuoles). Fig 5A shows that even though proteins from membrane organelles have a more positively charged N-terminus than cytosolic proteins, both groups are more positively charged up to their 50^th^ amino acid position. When the mitochondrial proteins were removed, the distribution of positive charges changed considerably (Fig 5B). This change can be explained by the presence of mitochondrial targeting sequences (MTS) that direct proteins to the mitochondria and undoubtedly contribute to the high net charges observed in the N-termini of this group[29]. Similarly, nuclear localization signals (NLSs) usually contain repeated lysine and arginine residues and could contribute to the N-terminal net charge in the nucleolus, chromosome, nucleoplasm, nuclear envelope and ribosome groups, even though they are not necessarily located at the N-terminal (see next section).

Unarguably, the post-translational function has a profound influence on the protein charge distribution as observed in the structure of membrane proteins[17] and the contribution of the MTS to the N-terminal charge (Fig 5). Furthermore, ribosome interacting proteins tend to present dynamic N and C termini that are enriched with positively charged residues, which facilitates the RNA interaction[30]. However, there is no clear explanation for the specific charge distribution in a vast number of proteins (i.e., cytoplasmic proteins). Taken together, these data suggested that while not all subcellular localizations showed an average positively charged N-termini, this feature was present in diverse organelles and proteins with different functional roles.

### N-terminal net charge does not correlate with the different parameters of the protein biology

While many proteins have positively charged stretches at their N and C-termini, not all proteins have net positive charges in this area. Therefore, we attempted to find a consistent correlation between the net charge of the first 30 amino acid residues of *S*. *cerevisiae* proteins and different aspects of protein synthesis and stability, such as the elongation rate, mRNA half-life or protein molecules per cell. The results of the correlation analysis are shown in S1 Table. Some analyses resulted in statistically significant P-values (< 0.005), but the squares of the correlation coefficient (r^2^) were all very small. For example, the best correlation value, i.e., a negative correlation between the net charge and the ORF length, had a P-value < 0.0001 with r^2^ = 0.01124, indicating that only 44 of the 4,448 analyzed proteins would show net charge values that vary with the protein size. Another significant negative correlation was found between the net charge and the total elongation time (P = 0.0002) and had even lower r^2^ values (0.0036). In conclusion, it was not possible to draw any functional hypotheses from these analyses. One possibility is that the correlation analysis is too stringent to detect co-variations that are likely not linear (see next sections) (S1 Table). Because positive charges are related to slower translation rates[4] and translation rates are related to co-translational protein folding[5,31], we investigated whether the N-terminal positive net charges are associated with chaperone recruitment. The *S*. *cerevisiae*’s chaperone interaction database[32] was used to analyze the average net charge of each chaperone’s clients. Because proteins with 300 amino acids or less tend to fold without the aid of chaperones while proteins with more than 300 amino acids usually need chaperone assistance[33], we also separated our database based on the protein length. Our analysis showed that both groups displayed the same distribution of N-terminal positive charges, suggesting that the protein charge is not an important factor in co-translational chaperone recruitment.

### N-terminal net charge correlates with ribosome occupancy

**Fig 6:**
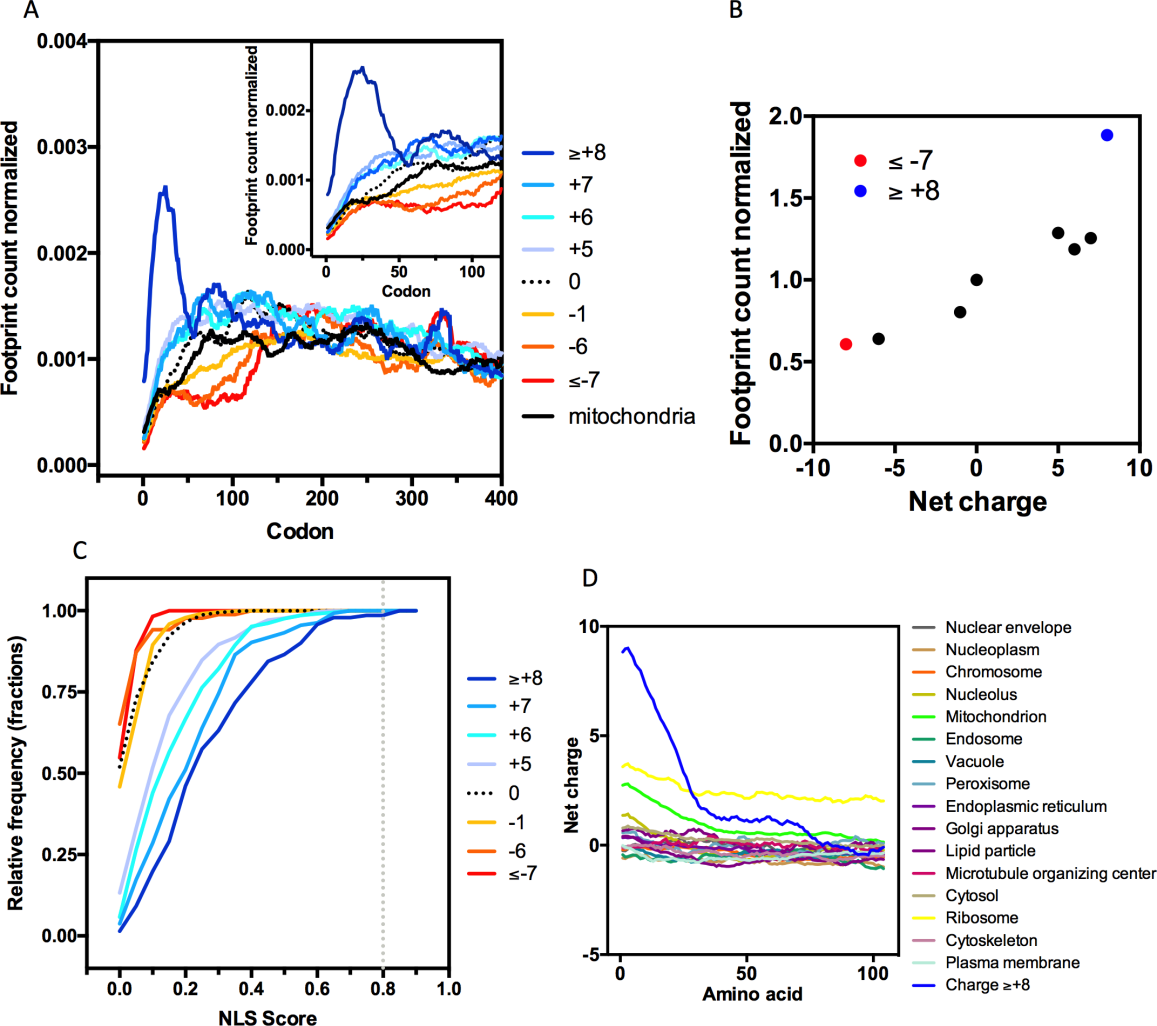
Ribosome profiling analyses of *S*. *cerevisiae* proteins grouped according to the net charge of the first 30 residues. **A.** Using the net charge of the first 30 residues, the *S*. *cerevisiae* proteome was subdivided into the following eight categories: proteins with a net charge ≥+8 (144 proteins), +7 (134 proteins), +6 (208 proteins), +5 (348 proteins), 0 (898 proteins), -1 (810 proteins), -6 (90 proteins) and ≤-7 (117 proteins). Mitochondrial proteins (414 proteins) were also included in this analysis. Ribosome profiling analysis was performed using data generated by the Brown laboratory[34]. Normalization was performed by dividing the number of reads at each position by the total number of reads in each gene. **B.** The area under the curve from codon 0 to codon 90 (corresponding to the first 30 amino acids) from panel A was calculated for each group of proteins and divided by the area under the curve for a net charge of 0. It is possible to observe a slight increase in the number of reads with positive net charges; however, for net charges greater than +8, it is possible to observe almost twice as many reads compared to those with a net charge of 0. **C.** The relative frequency of the NLS score was calculated by NucPred[35] for the first 30 amino acids of the proteins described in Panel A. A score threshold of 0.8 indicates a sensitivity of 0.30 and a specificity of 0.61. **D.** N-terminal net charge distribution in proteins from all sub-cellular localizations of *S*. *cerevisiae* compared with proteins with net charges ≥+8 (144) in the first 30 residues.

To establish a link between the charge distribution along the protein primary structure and the translation efficiency, we analyzed the translation rate at the N-terminal of proteins as a function of the nascent polypeptide charge. The effect of the N-terminal net charge on the ribosome occupancy was assessed using cycloheximide-free *S*. *cerevisiae* ribosome profiling data that were generated by the Brown laboratory[34]. The *S*. *cerevisiae* proteins were divided based on the net charge of their first 30 amino acid residues, and the ribosome occupancy was determined by plotting the number of reads at each position normalized to the average number of reads in the entire sequence of the corresponding mRNA. Fig 6A shows that the transcripts that code for positively charged protein segments tend to accumulate more ribosomes than the transcripts that code for negatively charged peptides. However, the effect of the nascent protein charge on the ribosome occupancy is clearly nonlinear (Fig 6B). Segments with net charges up to +5 occupy a number of ribosomes that is similar to those with neutral sequences, and only sequences with positive charges greater than +8 showed a two-fold increase in the ribosome density in their first codons. However, the mildly negatively charged segments showed a lower ribosome occupancy with values as small as -1, but the observed effect stabilized at charges below -6 (Fig 6B).

The GO analysis of the 144 proteins with N-terminal net charges ≥ +8 revealed that this group is enriched with nucleosome, ribosome and nucleolus components (S2 Table). Because most of these proteins have a nuclear localization, we investigated whether the NLS is present at the N-termini of these sequences and, therefore, is an important factor contributing to ribosome stalling. It is important to note that the NLS have diverse structures and compositions; therefore, prediction programs usually compute a probability score for putative motifs[35]. Fig 6C shows the cumulative distribution of the putative NLS determined by NucPred for the first 30 residues of proteins that belong to the charge groups analyzed in Fig 6A. Considering the recommended score threshold of 0.8 (sensitivity of 0.30 and specificity of 0.61)[35], only two proteins from the > +8 group presented an NLS at their N-terminal. Lowering the threshold to 0.5 did not substantially alter the number of putative NLS present in the positively charged group of proteins, excluding the possibility that the presence of an NLS accounts for the stalling of ribosomes, which is shown in Fig 6A. A mitochondrial targeting signal is also not common in the > +8 group, as shown in Fig 6A. Only six proteins had an MTS at their N-terminal, and the average ribosomal occupancy in the > +8 group was the same regardless of whether these proteins were excluded. Moreover, when we analyzed the ribosomal profiling of all yeast mitochondrial proteins (n = 414), the result was very similar to that in proteins with a 0 net charge at the N-termini (Fig 6A, compare dashed line with black line). As shown in Fig 6D, it becomes clear that the group of proteins with N-terminal net charges ≥ +8 belong to an exceptional set of proteins with net-charge values that are much higher than those in any other group that is created by cellular localization (Fig 6D).

We also analyzed the presence of the MTS and NLSs in the 2,307 super-charged proteins with net charges ≥ +14 located at any position of the protein in all organisms (Fig 1). Only 1% (25 proteins) were mitochondrial proteins. In contrast, we observed that NLSs are more common in the super-charged proteins than in a random group of yeast protein (S3 Fig), and approximately half of the NLSs with probability scores ≥ 0.8 were within the 60 residues that contained the supercharged stretch. Therefore, the super-charged proteins are enriched with NLSs, but the signal alone is not responsible for the particular high charge in this group (S3 Fig, compare blue symbols with red symbols).

Because most of the basic stretches at the N-terminal were within the range in which no important effect on translation efficiency was observed, the association between a physiological ramp and positively charged sequences, as proposed by Tuller *et al*.[16], may not be correct. Our conclusion is consistent with other work that has challenged this hypothesis. For example, studies suggest that cycloheximide influences ramp formation[19], and more recently, it has been observed that the ribosome profile of the polysomal fraction of the translating ribosomes has no ramp[7]. Nevertheless, it is clear that highly charged negative or positive peptides can modulate the ribosome occupancy (Fig 6). Moreover, rather than organizing the translation process, the important delay that is observed in ribosome movements with extremely positively charged proteins may signal errors, thereby reducing the translation efficiency. This delay could be one of the selection forces that act against extremely positively charged protein segments in the proteomes of different organisms (Fig 1).

### Positively charged protein segments are enriched in monosomes

Translating ribosomes exist in the following two different populations: monosomes, when a single ribosome is bound to the mRNA, and polysomes, when two or more ribosomes are bound to the mRNA. Polysomes are usually regarded as the active fraction of the ribosomal pool, but a recent study demonstrated that monosomes also actively translate and are responsible for processing transcripts that have initiation rates that are slower than the elongation and termination rates[7]. This condition was consistently found in ORFs shorter than 590 nt, in which the initiation time usually exceeds the elongation time; therefore, termination occurs before a second ribosome can initiate the translation. This condition was also present in longer ORFs, in which other factors may contribute to extending the initiation time, such as the presence of upstream open reading frames (uORFs)[7]. Polysomes, however, are formed in long transcripts with fast initiation rates.

**Fig 7:**
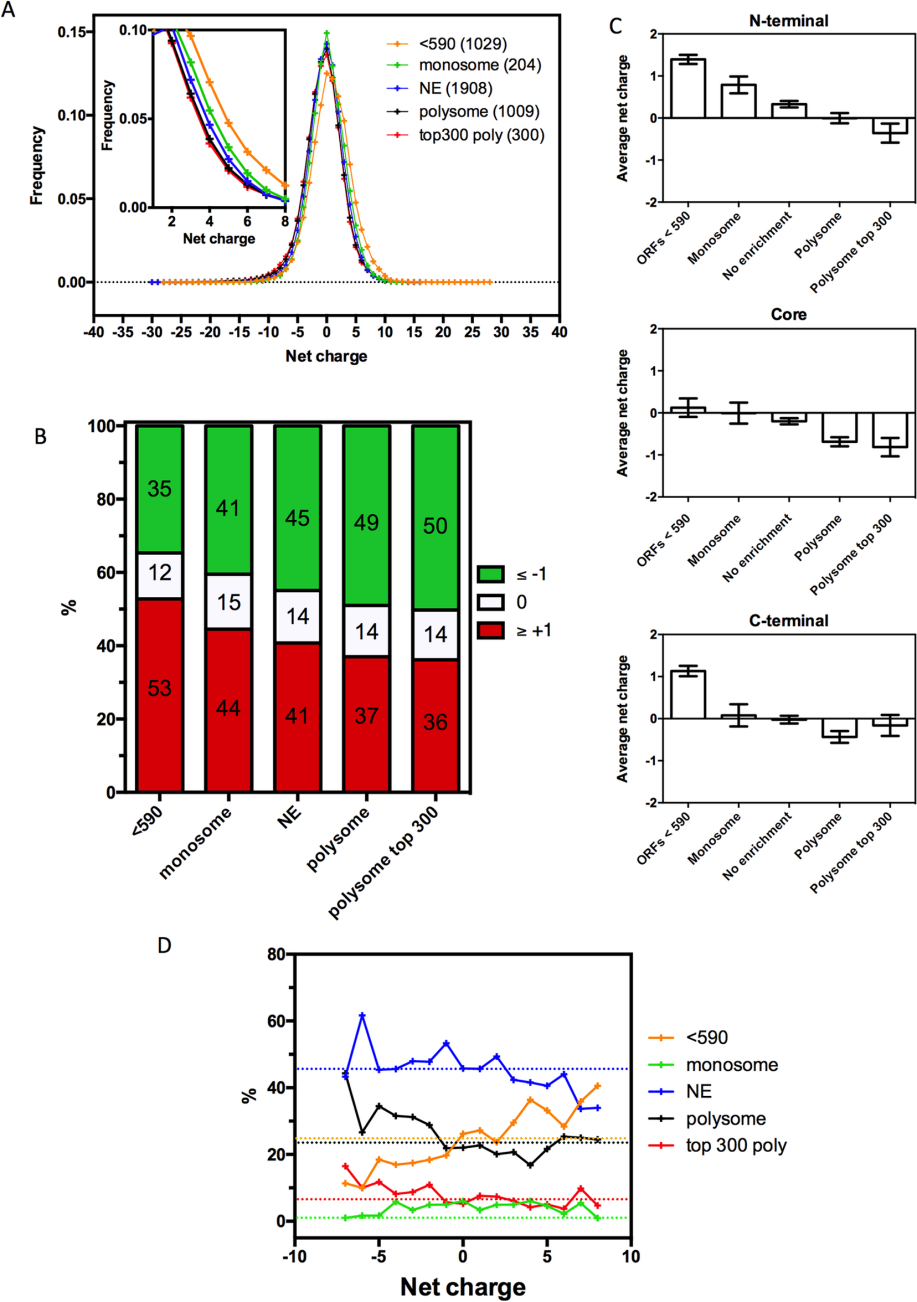
Positively charged N-termini are associated with monosomal translation. **A.** Net charge frequency histogram of differentially translated proteins in *S*. *cerevisiae*. Proteins were grouped based on their Monosome:Polysome scores[7] into “ORFs < 590” (1027 proteins, orange line), “Monosome enriched” (204 proteins, green line), “No enrichment” (1908 proteins, blue line), “Polysome enriched” (1006 proteins, black line) and “Top 300 polysome” (300 proteins, red line). There is an increasing concentration of positive sequences from “Top 300 polysome” to “ORFs < 590” (inset). **B.** Frequency of net charges in different groups. “ORFs < 590” contains 53% positively charged sequences, 12% neutral sequences and 35% negatively charged sequences; “Monosome enriched” contains 44% positively charged sequences, 15% neutral sequences and 41% negatively charged sequences; “No enrichment” contains 41% positively charged sequences, 14% neutral sequences and 45% negatively charged sequences; “Polysome enriched” contains 37% positively charged sequences, 14% neutral sequences and 49% negatively charged sequences and “Top 300 polysome” contains 36% positively charged sequences, 14% neutral sequences and 50% negatively charged sequences. **C.** Average N-terminal (1^st^), core (130^th^) and C-terminal (last) net charges in each of the groups shown in panel B. There is an N-terminal positive charge enrichment in the groups “ORFs < 590”, “Monosome enriched” and “No enrichment” (from higher to lower); there are neutral sequences in the group “Polysome enriched” and a negative charge enrichment in the group “Top 300 polysome”. This pattern (increasing concentration of positive sequences from “Top 300 polysome” to “ORFs < 590”) is maintained in the core with a positive charge enrichment in “ORFs < 590”, neutral sequences in “Monosome enriched” and a negative charge enrichment in “No enrichment”, “Polysome enriched” and “Top 300 polysome” (from lower to higher). C-terminal analyses do not show the same pattern, even though there is a positive charge concentration in “ORFs < 590”, a neutral sequence preference in “Monosome enriched” and “No enrichment” and a negative charge enrichment in “Polysome enriched” and “Top 300 polysome”. **D.** Percentage of proteins in different Monosome:Polysome groups separated by their N-termini net charges. *S*. *cerevisiae’s* proteins were separated by their N-termini net charges into 16 groups, ranging from -7 to +8 (the first dot, from left to right, represents proteins with N-termini net charges of -7 and below, while the last dot represents proteins with net charges of +8 and higher), and then, the proteins were separated into the Monosome:Polysome groups. Their Monosome:Polysome group distribution percentage was calculated and plotted. Dotted lines represent the percentage of all *S*. *cerevisiae’s* proteins in the Monosome:Polysome groups.

Because the ribosome occupancy analysis (Fig 6) suggested that the nascent polypeptide charge can affect the translation rate, we investigated whether the charged stretches could be related to monosomal or polysomal translation. To answer this question, we analyzed *S*. *cerevisiae* proteins that were classified according to their distribution in the monosomal or polysomal fractions as previously determined experimentally as follows[7]: “ORFs <590”, with proteins shorter than 590 nt (<590); “Monosome enriched”, with proteins longer than 590 nt but mainly found in the monosomal fraction; “No enrichment” (NE), with proteins equally found in both groups; and “Polysome enriched”, with proteins mainly translated by polysomes. The net charge of the first 30-amino-acid stretches was calculated, and the frequency of the distribution of these sequences is shown in Fig 7A. A shift toward a more basic net charge was observed in the “ORFs<590” and “Monosome enriched” groups compared to the “No enrichment category” (Fig 7A, 7A inset and 7B). Then, the average net charge of each group was analyzed with a focus on the N-terminal, core and C-terminal segments of each group of proteins (Fig 7C). It is clear that the monosome-enriched proteins had a higher N-terminal net charge (Monosome vs. Polysome, P < 0,05, **) and that the polysome-enriched proteins had a lower, even negative, N-terminal net charge (Fig 7C). This result indicates that proteins with positively charged N-termini are over-represented in the monosomal translation pool (groups “ORFs<590” and “Monosome enriched”), while negatively charged stretches are more abundant in the polysomal translation pool.

Fig 6B shows that the ribosome occupancy is more affected by the high values of the N-terminal net-charge. This observation prompted us to analyze the distribution of the different charge values in the monosomal and polysomal groups (Fig 7D). Proteins with positively charged N-termini are over represented in the <590 nt monosomal fractions (orange line), while the negatively charged N-termini tend to be translated by polysomes (black and red lines). It should be noted, however, that the monosome group formed by mRNAs with more than 590 nts is not enriched with proteins with highly basic N-termini (green line). Heyer and Moore noticed that this group is mainly populated by regulatory proteins with low expression levels. Some sequences contain uORFs that decrease the downstream reinitiating efficiency. However, for other sequences, it was not possible to find a simple explanation for the extremely long initiation time. Here, we show that a high concentration of basic charges cannot account for this effect, even though it could play a role together with other factors, such as the codon arrangement.

The presence of more proteins with highly positive N-termini in the ORFS <590 nt monosomal group is not a direct consequence of the N-terminal charge because the elongation and termination of sequences of this size are typically faster than the initiation time[7]. Moreover, the GO analysis of this group showed an enrichment in ribosomal and mitochondrial proteins (S3 Table), suggesting structural reasons for the presence of more positively charged sequences in the ORFS <590 nt group. However, we observed that the polysome and polysome top300 groups are even more enriched with ribosome components; however, in this high throughput mode of translation, sequences with neutral or negatively charged N-termini are more abundant than proteins with basic segments (Fig 7D). This distribution may reflect a selection for a rapid translation initiation and efficient elongation on polysomes, while short proteins, which are usually translated by monosomes, allow longer initiation or elongation times due to the basic polypeptides.

## Discussion

Our study shows that, universally, there are more negatively charged sequences than positively charged sequences (Fig 1). This difference is even greater in extremely charged sequences (charges greater than 14), suggesting that there is a strong evolutionary pressure against super positively charged proteins. Consistently with previously published works, we observed that the N- and C-termini of proteins tend to be enriched with positive stretches (Figs 2, 3, 4, 5, S1 and S2). Only a part of this distribution can be explained by the presence of localization signals at their N-termini or functional and structural reasons. Even though there was no substantial correlation between the N-terminal charge and certain aspects of translation (S1 Table), the super positively charged proteins were indeed able to significantly affect the translational speed (Fig 6). In fact, proteins with super positive N-termini are enriched with functions that are related to nucleic acid binding, such as ribosomal proteins and histones. This finding indicates that proteins that contain very positive stretches for a specific functional reason, such as nucleic acid binding and stabilization, may have to endure a lower translational efficiency. Since there is a translational stall in the synthesis of these positive proteins, it is possible that these proteins might require chaperone assistance to complete their folding or protect themselves from quality-control mechanisms, but no evidence for this hypothesis was observed. Finally, the N-terminal charge was compared across monosomal and polysomal populations. A positive charge and slow translation rates are suggested to aid polysomal translation because these factors can prevent ribosome jamming and collisions[16], but we observed an opposite trend (Fig 7). Positive N-termini appear to be related to monosomal translation (groups “ORFs > 590” and “Monosome enriched”), which is associated with slow initiation rates, while neutral or negative stretches are more common in proteins that are translated by polysomes, in which fast initiation and elongation rates are favored. The presence of basic peptides in short proteins that are translated by monosomes may be less disruptive than that in long proteins, in which a sudden drop in the translation speed may cause jams and abort translation. This analyses suggest that the translational efficiency may be one of the factors that shape the charge composition and distribution along the primary structures of the proteins.

## Methods

### Net charge and individual charge calculation

To calculate the net charge in a given sequence, a program that is able to screen the primary sequence of a given protein and attribute different values to different amino acids was developed. To determine the charge values for the amino acid residues, we considered the contribution of individual p*K*a values to the Henderson-Hasselbach equation at pH 7.4 (S1 Fig). The final peptide net charge was determined by summing each amino acid charge[36,37]. The following charge values were obtained: lysine = +0.999 (rounded value = + 1); arginine = +1.000; histidine = +0.048 (rounded value = 0); glutamic acid = -0.999 (rounded value = -1); aspartic acid = -1.000; cysteine = -0.085 (rounded value = 0) and all other amino acids = 0.000. The N- and C-termini were attributed the charges +0.996 and -1.000, respectively. After the charge attribution, the program calculated the net charge of every 30-amino-acid window in the primary sequence. The distribution of the protein fragments’ net charge from the *S*. *cerevisiae* proteome was determined considering charge values with 3 decimal places. The result was compared with a simpler calculation, which considered rounded charge values and disregarded the C- and N-terminal contributions (S1 Fig). Because the distributions were very similar, the rounded values were used in all other calculations.

To calculate the individual charge distribution, we developed a program that is able to screen the primary sequence of a given protein and attribute the same values as the last program, but this program provides the individual charges of the first and last 30 amino acids of the primary sequence.

### Protein database

The database SwissProt from uniprot.org was used as our source of primary sequences. The advanced search option (reviewed:yes) was used to select all proteins from SwissProt. Advanced search options for *H*. *sapiens* (reviewed:yes AND organism:"Homo sapiens (Human) [9606]"), *D*. *melanogaster* (reviewed:yes AND organism:"Drosophila melanogaster (Fruit fly) [7227]"), *C*. *elegans* (reviewed:yes AND organism:"Caenorhabditis elegans [6239]"), *A*. *thaliana* (reviewed:yes AND organism:"Arabidopsis thaliana (Mouse-ear cress) [3702]") and *S*. *cerevisiae* (reviewed:yes AND organism:"Saccharomyces cerevisiae (strain ATCC 204508 / S288c) (Baker's yeast) [559292]") were used as a source for the proteomes. For the analysis without the proteins that carry signal peptides, (NOT annotation:(type:signal 5840) AND reviewed:yes AND organism:"Homo sapiens (Human) [9606]") and (NOT annotation:(type:signal 5840) AND reviewed:yes AND organism:"Saccharomyces cerevisiae (strain ATCC 204508 / S288c) (Baker's yeast) [559292]" were used. For the subcellular localization analysis, proteins were separated according to their Gene Ontology ID (GO:0005635 nuclear envelope, GO:0005654 nucleoplasm, GO:0005694 chromosome, GO:0005730 nucleolus, GO:0005739 mitochondrion, GO:0005764 lysosome, GO:0005768 endosome, GO:0005773 vacuole, GO:0005777 peroxisome, GO:0005783 endoplasmic reticulum, GO:0005794 Golgi apparatus, GO:0005811 lipid particle, GO:0005815 microtubule organizing center, GO:0005829 cytosol, GO:0005840 ribosome, GO:0005856 cytoskeleton, and GO:0005886 plasma membrane).

### Heat map

To create the positive/negative heat maps displayed in this paper, Orange 3.2 (orange.biolab.si) software was used. After downloading the primary sequences and calculating the charge, our output files were uploaded onto Orange 3.2, and the “Heat Map” option was selected from the “Visualize” menu. The first one hundred net-charge values were used for the N-terminal analyses, and the last one hundred net-charge values were used for the C-terminal analyses. For the core residue analyses, the 130^th^ to 229^th^ net-charge values were utilized.

### Chaperones

To analyze the correlations between the co-translational folding and the N-terminal net charges, the *S*. *cerevisiae* proteome interaction database was used[32]. The *S*. *cerevisiae* proteins were separated based on their chaperone interactions, and their first 100 average net-charge values were plotted. Chaperones with 30 clients or less were not included.

### Ribosome profiling data

To analyze the correlations between the ribosome density and the net charges, *S*. *cerevisiae* ribosome profiling data were used[33]. The data were analyzed as described previously[14]. Briefly, the data were downloaded from GEO, and the adaptors (CTGTAGGCACCATCAAT) were trimmed. The trimmed FASTA sequences were aligned to *S*. *cerevisiae* ribosomal and noncoding RNA sequences to remove the rRNA reads. The unaligned reads were aligned to the *S*. *cerevisiae* S288C genome, which is deposited in the Saccharomyces genome database. First, any reads that mapped to multiple locations were removed. Then, the reads were aligned to the *S*. *cerevisiae* coding sequence database, allowing two mismatches per read. Genes with <50% of positions covered were eliminated. To analyze the N-terminal correlation with the ribosome density, the *S*. *cerevisiae* proteins were separated into the following eight categories based on their first net charge: ≥+8 (first net charges from +8 to +13), “+7”, “+6”, “+5”, “0”, “-1”, “-6” and ≤-7 (first net charges from -7 to -15). Then, the first averaged ribosome density of each group was plotted.

### Nuclear localization signals

To evaluate whether N-termini with a net charge ≥ +8 were enriched with NLSs, we individually analyzed the N-terminal region (first 30 amino acids) of all 144 proteins with N-terminal charges ≥ +8 using NucPred[35]. To analyze whether peptides with a net charge ≥ +14 were enriched with NLSs, we used a program that was able to identify these highly charged peptides and provide their sequences plus the next thirty amino acids (totalizing sixty amino acids) as the output; we then used the NucPred batch predictor[35] on these data.

### Monosome vs polysome

To compare the net-charge values between monosomal or polysomal translation, the Monosome:Polysome scores were utilized[7]. Based on these data, the *S*. *cerevisiae* proteins were separated into five categories (ORF<590, monosome enriched, no enrichment, polysome enriched and polysome top 300). After the protein categorization, the average first net charge in each category was calculated for the N-terminal net charge, the average 121 net charge for the core residues net charge, and the C-terminal net charge.

## Supporting information

S1 Fig C-terminal positive charge concentrations in different subcellular locations. Heat map of the *H*. *sapiens*, *D*. *melanogaster*, *C*. *elegans*, *A*. *thaliana* and *S*. *cerevisiae* proteomes showing the C-terminal average net charge distribution (net charges from amino acids 1 to 30 until 100 to 129) divided into 17 different subcellular locations. Red tiles represent the positively charged sequences, black tiles represent the neutral sequences and green tiles represent the negatively charged sequences. Differently from the N-terminal segments, the C-terminal positive charge concentrations do not appear to be conserved among most subcellular locations, even though most subcellular locations show some degree of a C-terminal positive charge.
(TIFF)

S2 Fig Average ribosomal protein N-terminal charge distribution. Heat map of the *H*. *sapiens*, *D*. *melanogaster*, *C*. *elegans*, *A*. *thaliana* and *S*. *cerevisiae* ribosomal proteins showing the N-terminal average net charge distribution (net charges from amino acids 1 to 30 until 100 to 129). Red tiles represent the positively charged sequences, black tiles represent the neutral sequences and green tiles represent the negatively charged sequences. Even though these groups of proteins are already very positively charged, their N-termini still follow the pattern of positive charge concentrations.
(TIFF)

S3 Fig Relative frequency of NLS scores in super-charged proteins (≥ +14). Relative frequency of NLS scores in a control group (2,000 random proteins from *S*. *cerevisiae*, black line), the full-length 2,307 super-charged proteins from all organisms (blue line) and the stretches (30 amino acids with a net charge ≥ +14 plus the subsequent 30 aa) from the super-charged proteins (red line). The full-length super-charged proteins present higher NLS scores than both the control and stretches ≥ +14. This finding indicates that segments of the super positive proteins, rather than other stretches ≥ +14, contributed to their high NLS scores likely because most of these proteins are found in the nucleus.
(TIFF)

S4 Fig Different net charge calculations in *S*. *cerevisiae*. **A.** Net-charge frequency histogram of the amino-acid segments of all 6,721 proteins from the *S*. *cerevisiae’s* proteome using the values from Table A (in figure, red line) and Table B (in figure, blue line). When Table B values were used, we can observe a slight increase in the negatively charged peptides (upper-right inset). **B.** Net charge ratio between the negative and positive sequences shows a steep increase around charge 14 in both calculations. **C.** Heat map of the *S*. *cerevisiae* proteome with both calculations shows very similar net charge distributions. Red tiles represent the positively charged sequences, black tiles represent the neutral sequences and green tiles represent the negatively charged sequences. The first tile was omitted since the value was very positive (+1.7) because of the contribution of N-term (+1) of each protein.
(TIFF)

S1 Table N-terminal net charge correlation with different parameters of protein biology. Net charge values from the first 30 amino acids from *S*. *cerevisiae* correlation with mRNA ORF length (nucleotides), mRNA five prime UTR length (base pairs), translation initiation time (milliseconds), total elongation time (milliseconds), ratio of initiation (initiation time/total elongation time), elongation rate (codons per second), transcription rate (mol per minutes), number of ribosomes per mRNA molecule, ribosome density (the number of ribosomes per 100 codons), total mRNA half life (minutes), protein molecules per cell and total protein molecules. After being subjected to D’agostino and Pearson omnibus normality test to check if they came from a Gaussian distribution, values were analyzed with Pearson or Spearman correlation test.
(DOCX)

S2 Table GO analysis of highest positively charged N-terminal proteins. GO analysis of the 144 *S*.*cerevisiae’s* proteins with N-terminal net charges ≥ +8.
(XLSX)

S3 Table GO analysis of different Monosome:Polysome groups. GO analysis of all *S*.*cerevisiae’s* proteins separated by their Monosome:Polysome scores.
(XLSX)

## References

[1] Hershey JWB, Sonenberg N, Mathews MB. Principles of Translational Control: An Overview. Cold Spring Harb Perspect Biol. 2012;4. 10.1101/cshperspect.a011528 PMC3504442 23209153

[2] Komar AA. A pause for thought along the co-translational folding pathway. Trends Biochem Sci. 2009;34:16–24. 10.1016/j.tibs.2008.10.002 18996013

[3] Yu C-H, Dang Y, Zhou Z, Wu C, Zhao F, Sachs MS, et al Codon Usage Influences the Local Rate of Translation Elongation to Regulated Co-translational Protein Folding Mol Cell. 2015;59:744–54. 10.1016/j.molcel.2015.07.018 26321254 PMC4561030

[4] Lu J, Deutsch C. Electrostatics in the Ribosomal Tunnel Modulate Chain Elongation Rates. J Mol Biol. 2008;384:73–86. 10.1016/j.jmb.2008.08.089 18822297 PMC2655213

[5] Thanaraj TA, Argos P. Ribosome-mediated translational pause and protein domain organization. Protein Sci. 1996;5:1594–612. 10.1002/pro.5560050814 8844849 PMC2143486

[6] Inada T. The Ribosome as a Platform for mRNA and Nascent Polypeptide Quality Control. Trends Biochem Sci. 2017;42:5–15. 10.1016/j.tibs.2016.09.005 27746049

[7] Heyer EE, Moore MJ. Redefining the Translational Status of 80S Monosomes. Cell. 2016;164:757–69. 10.1016/j.cell.2016.01.003 26871635

[8] Moore PB, Steitz TA. The structural basis of large ribosomal subunit function. Annu Rev Biochem. 2003;72:813–50. 10.1146/annurev.biochem.72.110601.135450 14527328

[9] Fedyukina DV, Cavagnero S. Protein folding at the exit tunnel. Annu Rev Biophys. 2011;40:337–59. 10.1146/annurev-biophys-042910-155338 21370971 PMC5807062

[10] Lu J, Kobertz WR, Deutsch C. Mapping the electrostatic potential within the ribosomal exit tunnel. J Mol Biol. 2007;371:1378–91. 10.1016/j.jmb.2007.06.038 17631312

[11] Ingolia NT, Brar GA, Rouskin S, McGeachy AM, Weissman JS. The ribosome profiling strategy for monitoring translation in vivo by deep sequencing of ribosome-protected mRNA fragments. Nat Protoc. 2012;7:1534–50. 10.1038/nprot.2012.086 22836135 PMC3535016

[12] Charneski CA, Hurst LD. Positively charged residues are the major determinants of ribosomal velocity. PLoS Biol. 2013;11:e1001508 10.1371/journal.pbio.1001508 23554576 PMC3595205

[13] Artieri CG, Fraser HB. Accounting for biases in riboprofiling data indicates a major role for proline in stalling translation. Genome Res. 2014;24:2011–21. 10.1101/gr.175893.114 25294246 PMC4248317

[14] Requião RD, de Souza HJA, Rossetto S, Domitrovic T, Palhano FL. Increased ribosome density associated to positively charged residues is evident in ribosome profiling experiments performed in the absence of translation inhibitors. RNA Biol. 2016;13:561–8. 10.1080/15476286.2016.1172755 27064519 PMC4962802

[15] Berezovsky IN, Kilosanidze GT, Tumanyan VG, Kisselev LL. Amino acid composition of protein termini are biased in different manners. Protein Eng. 1999;12:23–30. 10065707 10.1093/protein/12.1.23

[16] Tuller T, Veksler-Lublinsky I, Gazit N, Kupiec M, Ruppin E, Ziv-Ukelson M. Composite effects of gene determinants on the translation speed and density of ribosomes. Genome Biol. 2011;12:R110 10.1186/gb-2011-12-11-r110 22050731 PMC3334596

[17] Charneski CA, Hurst LD. Positive charge loading at protein termini is due to membrane protein topology, not a translational ramp. Mol Biol Evol. 2014;31:70–84. 10.1093/molbev/mst169 24077849

[18] Tuller T, Carmi A, Vestsigian K, Navon S, Dorfan Y, Zaborske J, et al An Evolutionarily Conserved Mechanism for Controlling the Efficiency of Protein Translation. Cell. 2010;141:344–54. 10.1016/j.cell.2010.03.031 20403328

[19] Gerashchenko MV, Gladyshev VN. Translation inhibitors cause abnormalities in ribosome profiling experiments. Nucleic Acids Res. 2014;42:e134 10.1093/nar/gku671 25056308 PMC4176156

[20] Weinberg DE, Shah P, Eichhorn SW, Hussmann JA, Plotkin JB, Bartel DP. Improved Ribosome-Footprint and mRNA Measurements Provide Insights into Dynamics and Regulation of Yeast Translation. Cell Rep. 2016;14:1787–99. 10.1016/j.celrep.2016.01.043 26876183 PMC4767672

[21] Thurlkill RL, Grimsley GR, Scholtz JM, Pace CN. pK values of the ionizable groups of proteins. Protein Sci. 2006;15:1214–8. 10.1110/ps.051840806 16597822 PMC2242523

[22] Pace CN, Grimsley GR, Scholtz JM. Protein ionizable groups: pK values and their contribution to protein stability and solubility. J Biol Chem. 2009;284:13285–9. 10.1074/jbc.R800080200 19164280 PMC2679426

[23] Messerli MA, Amaral-Zettler LA, Zettler E, Jung S-K, Smith PJS, Sogin ML. Life at acidic pH imposes an increased energetic cost for a eukaryotic acidophile. J Exp Biol. 2005;208:2569–79. 10.1242/jeb.01660 15961743

[24] Zalucki YM, Power PM, Jennings MP. Selection for efficient translation initiation biases codon usage at second amino acid position in secretory proteins. Nucleic Acids Res. 2007;35:5748–54. 10.1093/nar/gkm577 17717002 PMC2034453

[25] Kozak M. Downstream secondary structure facilitates recognition of initiator codons by eukaryotic ribosomes. Proc Natl Acad Sci U S A. 1990;87:8301–5. 2236042 10.1073/pnas.87.21.8301 PMC54943

[26] Björnsson A, Mottagui-Tabar S, Isaksson LA. Structure of the C-terminal end of the nascent peptide influences translation termination. EMBO J. 1996;15:1696–704. 8612594 PMC450081

[27] Mottagui-Tabar S, Björnsson A, Isaksson LA. The second to last amino acid in the nascent peptide as a codon context determinant. EMBO J. 1994;13:249–57. 8306967 10.1002/j.1460-2075.1994.tb06255.x PMC394799

[28] Gene Ontology Consortium. Gene Ontology Consortium: going forward. Nucleic Acids Res. 2015;43:D1049–56. 10.1093/nar/gku1179 25428369 PMC4383973

[29] Schmidt O, Pfanner N, Meisinger C. Mitochondrial protein import: from proteomics to functional mechanisms. Nat Rev Mol Cell Biol. 2010;11:655–67. 10.1038/nrm2959 20729931

[30] Brodersen DE, Clemons WM, Carter AP, Wimberly BT, Ramakrishnan V. Crystal structure of the 30 S ribosomal subunit from Thermus thermophilus: structure of the proteins and their interactions with 16 S RNA. J Mol Biol. 2002;316:725–68. 10.1006/jmbi.2001.5359 11866529

[31] O'Brien EP, Ciryam P, Vendruscolo M, Dobson CM. Understanding the influence of codon translation rates on cotranslational protein folding. Acc Chem Res. 2014;47:1536–44. 10.1021/ar5000117 24784899

[32] Gong Y, Kakihara Y, Krogan N, Greenblatt J, Emili A, Zhang Z, et al An atlas of chaperone-protein interactions in Saccharomyces cerevisiae: implications to protein folding pathways in the cell. Mol Syst Biol. 2009;5:275 10.1038/msb.2009.26 19536198 PMC2710862

[33] Hartl FU, Bracher A, Hayer-Hartl M. Molecular chaperones in protein folding and proteostasis. Nature. 2011;475:324–32. 10.1038/nature10317 21776078

[34] Lareau LF, Hite DH, Hogan GJ, Brown PO. Distinct stages of the translation elongation cycle revealed by sequencing ribosome-protected mRNA fragments. Elife. 2014;3:e01257 10.7554/eLife.01257 24842990 PMC4052883

[35] Brameier M, Krings A, MacCallum RM. NucPred—predicting nuclear localization of proteins. Bioinformatics. 2007;23:1159–60. 10.1093/bioinformatics/btm066 17332022

[36] Patrickios CS, Yamasaki EN. Polypeptide amino acid composition and isoelectric point. II. Comparison between experiment and theory. Anal Biochem. 1995;231:82–91. 10.1006/abio.1995.1506 8678324

[37] Audain E, Ramos Y, Hermjakob H, Flower DR, Perez-Riverol Y. Accurate estimation of isoelectric point of protein and peptide based on amino acid sequences. Bioinformatics. 2016;32:821–7. 10.1093/bioinformatics/btv674 26568629 PMC5939969

