# Supplementary figures and images for: Protein charge distribution in proteomes and its impact on translation
Source: PLoS Comput Biol. 2017 May 22;13(5):e1005549. doi: 10.1371/journal.pcbi.1005549 (PMC5460897; doi:10.1371/journal.pcbi.1005549)

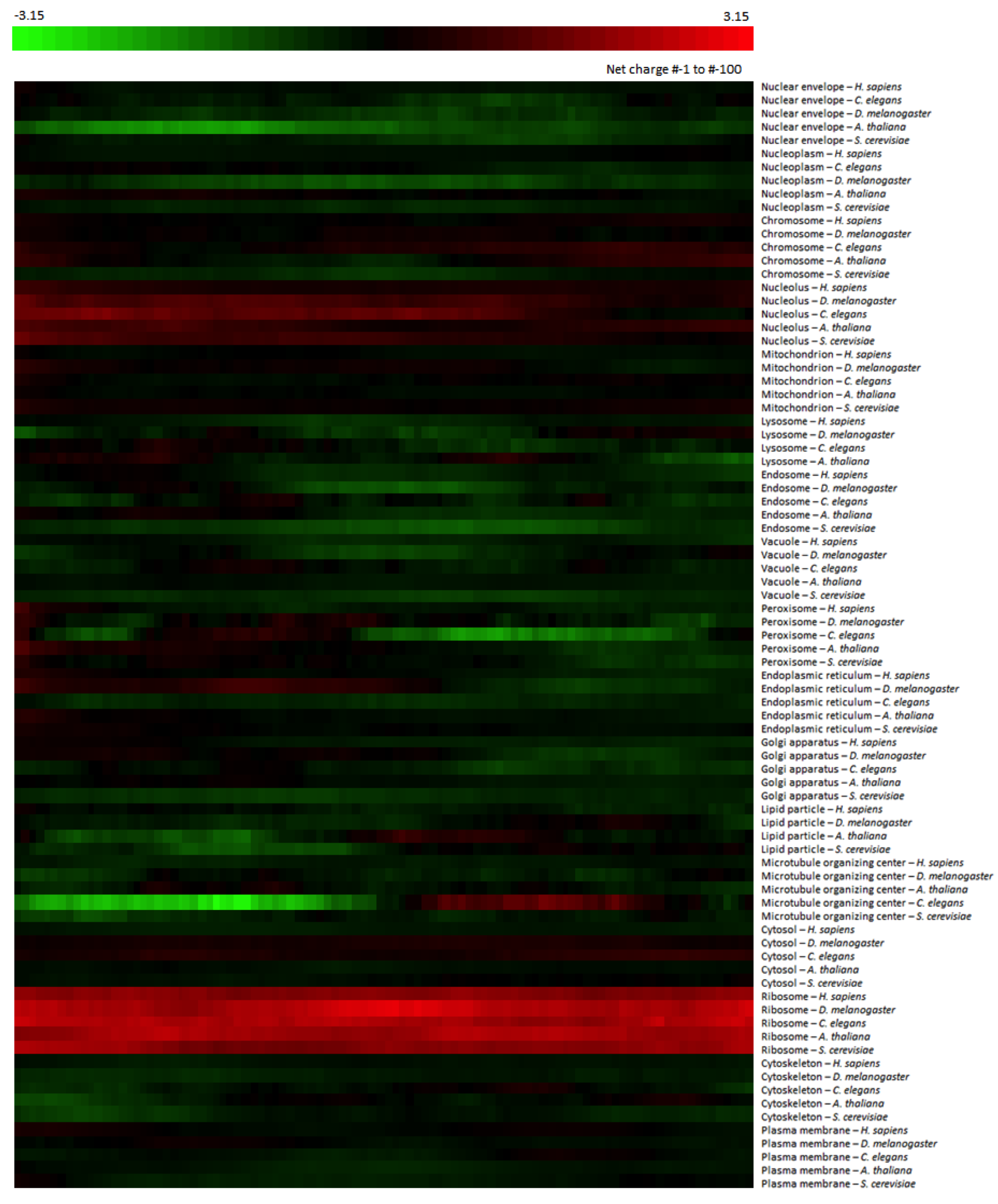

Supplement: S1 Fig — Heat map of the H. sapiens, D. melanogaster, C. elegans, A. thaliana and S. cerevisiae proteomes showing the C-terminal average net charge distribution (net charges from amino acids 1 to 30 until 100 to 129) divided into 17 different subcellular locations. Red tiles represent the positively charged sequences, black tiles represent the neutral sequences and green tiles represent the negatively charged sequences. Differently from the N-terminal segments, the C-terminal positive charge concentrations do not appear to be conserved among most subcellular locations, even though most subcellular locations show some degree of a C-terminal positive charge. (TIFF) [file pcbi.1005549.s001.tiff]

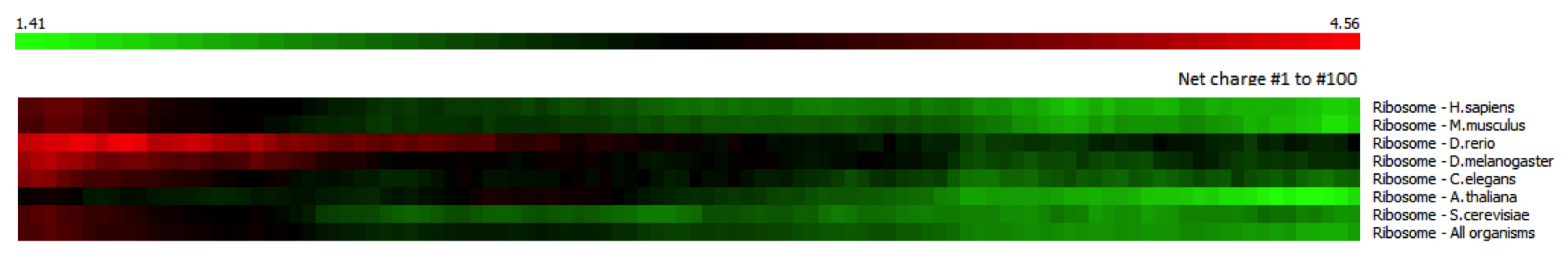

Supplement: S2 Fig — Heat map of the H. sapiens, D. melanogaster, C. elegans, A. thaliana and S. cerevisiae ribosomal proteins showing the N-terminal average net charge distribution (net charges from amino acids 1 to 30 until 100 to 129). Red tiles represent the positively charged sequences, black tiles represent the neutral sequences and green tiles represent the negatively charged sequences. Even though these groups of proteins are already very positively charged, their N-termini still follow the pattern of positive charge concentrations. (TIFF) [file pcbi.1005549.s002.tiff]

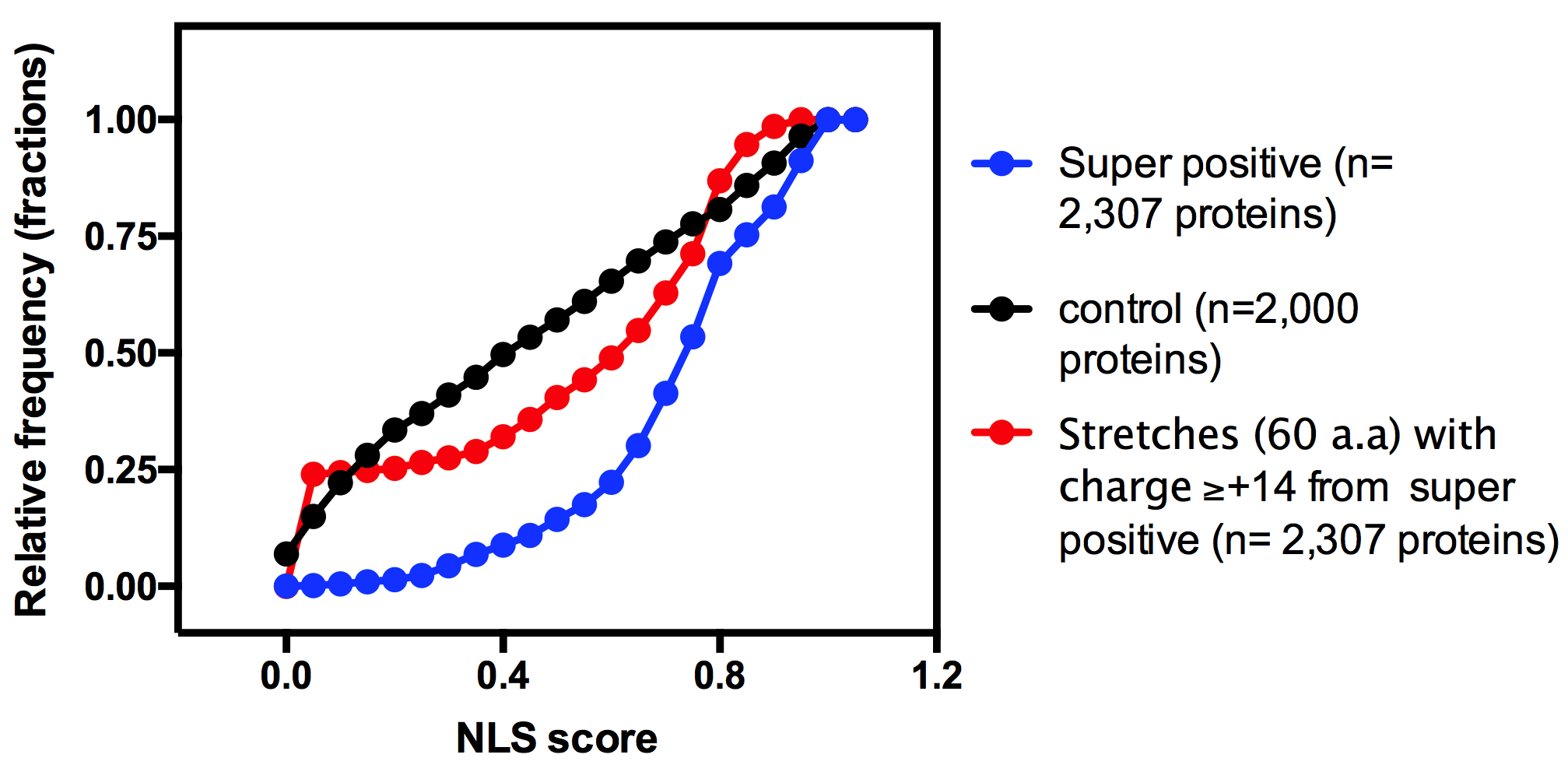

Supplement: S3 Fig — Relative frequency of NLS scores in a control group (2,000 random proteins from S. cerevisiae, black line), the full-length 2,307 super-charged proteins from all organisms (blue line) and the stretches (30 amino acids with a net charge ≥ +14 plus the subsequent 30 aa) from the super-charged proteins (red line). The full-length super-charged proteins present higher NLS scores than both the control and stretches ≥ +14. This finding indicates that segments of the super positive proteins, rather than other stretches ≥ +14, contributed to their high NLS scores likely because most of these proteins are found in the nucleus. (TIFF) [file pcbi.1005549.s003.tiff]

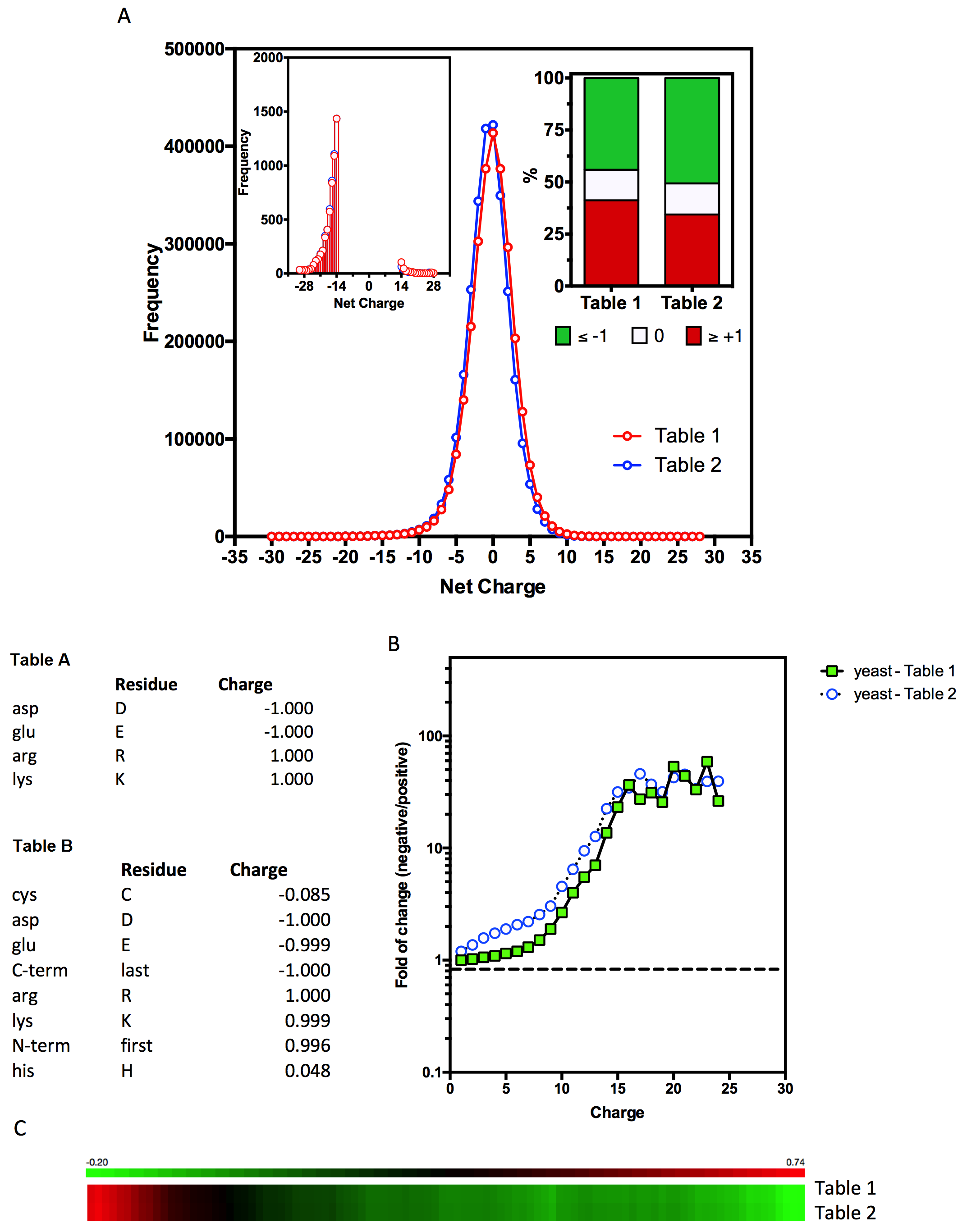

Supplement: S4 Fig — A. Net-charge frequency histogram of the amino-acid segments of all 6,721 proteins from the S. cerevisiae’s proteome using the values from Table A (in figure, red line) and Table B (in figure, blue line). When Table B values were used, we can observe a slight increase in the negatively charged peptides (upper-right inset). B. Net charge ratio between the negative and positive sequences shows a steep increase around charge 14 in both calculations. C. Heat map of the S. cerevisiae proteome with both calculations shows very similar net charge distributions. Red tiles represent the positively charged sequences, black tiles represent the neutral sequences and green tiles represent the negatively charged sequences. The first tile was omitted since the value was very positive (+1.7) because of the contribution of N-term (+1) of each protein. (TIFF) [file pcbi.1005549.s004.tiff]
