# Supplementary material for: Protein charge distribution in proteomes and its impact on translation
Source: PLoS Comput Biol. 2017 May 22;13(5):e1005549. doi: 10.1371/journal.pcbi.1005549 (PMC5460897; doi:10.1371/journal.pcbi.1005549)
Supplement: S1 Table — Net charge values from the first 30 amino acids from S. cerevisiae correlation with mRNA ORF length (nucleotides), mRNA five prime UTR length (base pairs), translation initiation time (milliseconds), total elongation time (milliseconds), ratio of initiation (initiation time/total elongation time), elongation rate (codons per second), transcription rate (mol per minutes), number of ribosomes per mRNA molecule, ribosome density (the number of ribosomes per 100 codons), total mRNA half life (minutes), protein molecules per cell and total protein molecules. After being subjected to D’agostino and Pearson omnibus normality test to check if they came from a Gaussian distribution, values were analyzed with Pearson or Spearman correlation test. (DOCX) [file pcbi.1005549.s005.docx]

**Supplemental table 1. N-terminal net charge correlation with different parameters of protein biology.** Net charge values from the first 30 amino acids from *S. cerevisiae* correlation with mRNA ORF length (nucleotides), mRNA five prime UTR length (base pairs), translation initiation time (milliseconds), total elongation time (milliseconds), ratio of initiation (initiation time/total elongation time), elongation rate (codons per second), transcription rate (mol per minutes), number of ribosomes per mRNA molecule, ribosome density (the number of ribosomes per 100 codons), total mRNA half life (minutes), protein molecules per cell and total protein molecules. After being subjected to D’agostino and Pearson omnibus normality test to check if they came from a Gaussian distribution, values were analyzed with Pearson or Spearman correlation test.

| Name | P Value | R | R^2^ | Number of values |
| --- | --- | --- | --- | --- |
| ORF Length | < 0.0001 | -0.1060 | 0.01124 | 4448 |
| Num. of Ribosomes | < 0.0001 | -0.09258 | 0.008572 | 1676 |
| Transcription Rate | < 0.0001 | 0.07522 | 0.005658 | 3321 |
| Total Half Life | 0.1797 | -0.01687 | 0.000284 | 2954 |
| Five prime UTR Length | 0.3918 | 0.004739 | 2.246e-005 | 3363 |
| Protein Molecules per Cell | 0.0029 | 0.04953 | 0.002453 | 3108 |
| Initiation Time | 0.1412 | -0.01816 | 0.0003300 | 3504 |
| Total Elongation Time | 0.0002 | -0.06033 | 0.003640 | 3504 |
| Ratio Init Elong | 0.4601 | -0.001694 | 2.869e-006 | 3504 |
| Ribosome Density | 0.0699 | 0.02495 | 0.0006227 | 3504 |
| Total Protein Molecules | 0.0318 | 0.03230 | 0.001043 | 3301 |
| Elong Rate | < 0.0001 | 0.08657 | 0.007494 | 3504 |
